# Editorial: Combinatorial Approaches to Enhance Anti-tumor Immunity: Focus on Immune Checkpoint Blockade Therapy

**DOI:** 10.3389/fimmu.2019.02083

**Published:** 2019-09-06

**Authors:** Patrik Andersson, Christian Ostheimer

**Affiliations:** ^1^Edwin L. Steele Laboratories, Department of Radiation Oncology, Harvard Medical School, Massachusetts General Hospital, Boston, MA, United States; ^2^Department of Microbiology, Tumor and Cell Biology, Karolinska Institutet, Stockholm, Sweden; ^3^Medical Science Division, Department of Oncology, Oxford Institute for Radiation Oncology, University of Oxford, Oxford, United Kingdom; ^4^Department of Radiation Oncology, Martin Luther University Halle-Wittenberg, Halle, Germany

**Keywords:** cancer, immunotherapy, radiotherapy, antiangiogenic therapy, immune checkpoint blockade (ICB), cancer vaccine

## Introduction

The advent of immunotherapy (IT), especially immune checkpoint blockers (ICBs), and its application in oncology has provided new hope for cancer patients. However, despite the rapid progress in the field of immunoncology, only a subset of patients currently benefit from these therapies. Many challenges remain to be resolved in order for IT to display optimal efficacy and good overall response rates in patients. First, many tumors have low tumor mutational burden (TMB), and therefore only produce limited antigens that can be recognized by endogenous T cells (1). Second, reduced antigen release or downregulation of antigen presentation machinery contributes to immune escape, leading to tumors with scarce numbers of infiltrating immune cells, indicating that reinvigoration of the pre-existing pool of anti-tumor T cells by ICBs may not be enough to induce tumor regression (2, 3). And third, even if the number and activity of T cells are successfully boosted by immunostimulatory therapies, the immunosuppressive tumor microenvironment (TME) restricts durable responses and contributes to treatment resistance (4). To overcome these limitations, new strategies are needed. Currently, several approaches exist where IT is combined with standard-of-care therapies, including radiotherapy (RT) and/or anti-angiogenic therapy (AAT) that are being evaluated in both preclinical and clinical settings. The aim of this article collection is to provide a comprehensive overview of recent developments and approaches in enhancing anti-tumor immunity with the focus on potential synergistic effects of RT and/or AAT with IT, ultimately supporting the rationale of combining IT with AAT and RT.

## Strategy 1: Increase Antigen Production, Release and Presentation

Recent findings indicate that high TMB is positively correlated to ICB responses across different types of tumors (5). DNA damaging therapies such as standard-of-care RT can be applied to induce reactive oxygen species (ROS), leading to immunogenic cell death and antigen release (6). Another strategy involves using RT at sublethal levels to induce mutations to increase antigens, which would aid in immune recognition (7, 8). However, standard dosing of RT could be immunosuppressive by direct effects on lymphocytes and dendritic cells (DCs) (9). In contrast, recent evidence suggests that stereotactic body radiation therapy increases T cell activity and reduces inhibitory stroma in tumors (Menon et al.). It has been demonstrated that tumor-derived exosomes successfully delivered double-stranded DNA and induced IFN-mediated T cell responses more efficiently in irradiated mice (10). Furthermore, through increased recruitment and activity of DCs, owing to RT-induced expression of vascular endothelial cell adhesion protein 1 on the endothelium and CXCL16 in tumor cells (11, 12), RT could also directly promote T cell activation and priming. RT has been shown to stimulate the production of type I interferons (IFNs), leading to increased number of CD8α+ tumor infiltrating DCs and subsequent boost in antigen presentation and T cell priming (13–15). Interestingly, selecting the optimal dose seems to be crucial to determine the anti-tumor response. For example, high-dose RT (20 Gy x 2) prevented beneficial production of type I IFNs by induction of Trex1, which degrades double-stranded DNA released by radiation-induced tumor cell death (16). Nevertheless, it will be important to assess the immune response to RT in individual patients/tumors, which has recently been reviewed elsewhere (17).

Additionally, the TME seems to play an important role in antigen presentation by regulating DC function. Jiang et al. found that high tumor cell-intrinsic expression of FASN (fatty acid synthase) led to increased lipid accumulation in DCs, which reduced their antigen presenting capacity in an ovarian cancer model. As reported, blocking FASN increased T cell infiltration, hence, it would be reasonable to speculate that ICBs may be rendered more effective.

## Strategy 2: Vaccination

Instead of strategies aiming to increase the antigenicity of tumors by killing tumor cells or increasing their mutational load, vaccines can be utilized to take advantage of pre-existing alterations in tumors. Vaccines come in different flavors, including whole tumor cell lysates, synthesized proteins or peptides, viral vectors expressing tumor antigens, or DC-based vaccines. Utilizing pulsed mature DC-based vaccines could potentially overcome some of the immunosuppressive cues, which otherwise could reduce vaccine efficacy by limiting DC migration, maturation and antigen presentation (18). Unfortunately, only limited benefits with therapeutic vaccine monotherapy have been observed clinically. Even if the vaccines themselves successfully circumvent antigen presentation and priming of T cells, downstream obstacles of immunosuppression could still remain. Metabolic reprogramming in the TME could play an essential role in immunosuppression. For example, by depleting tryptophan and producing kynurenine, indoleamine 2,3-dioxygenase (IDO) promotes the generation of immunosuppressive regulatory T cells (Tregs) and myeloid-derived suppressor cells (MDSCs) (19). Experimentally, Moreno et al., show that treatment of HPV+ tumors with immunometabolic adjuvants (such as IDO inhibitors) could induce a therapeutic benefit of an otherwise ineffective HPV-16 vaccine. However, as Eleftheriadis elaborates on in his opinion piece, IDO inhibitors have so far failed clinically and researchers are currently trying to understand why.

These preclinical and clinical lessons collectively suggest that combinatorial approaches could offer great clinical benefits to boost vaccines. Mougel et al. discuss the rationale for combining vaccines with AAT or ICBs to overcome tumor-employed immune escape mechanisms, with a focus on current clinical efforts. In addition, van Gulijk et al. provide an overview specifically on DC-based vaccines and strategies to combine with chemotherapy, RT and ICBs.

## Strategy 3: Increase T Cell Infiltration

Tumor tissues typically display limited number and heterogeneous distribution of T cells. Leukocyte infiltration is an active process that can be facilitated or hindered by the endothelium of blood vessels. Blood vessels are critical mediators of inflammation by providing a direct interface with which immune cells interact to gain access to tissues. Upon inflammatory cues, the endothelial cells lining the inner surface of blood vessels will express adhesion molecules and soluble mediators of leukocyte trafficking. In tumors, however, the immature nature of blood vessels can cause endothelial anergy, a state of lymphocyte tolerance characterized by repression of adhesion molecules, leading to failure of leukocyte trafficking (20–22). Klein provides a detailed review specifically on the tumor endothelium, with its implications for combination therapies using RT or IT. The endothelial-immune interface provides an opportunity for intervention, where AAT could be applied to increase the influx of anti-tumor immune cells. Strategies to normalize tumor vessels, with an overview on current preclinical and clinical efforts, and potential synergy with IT are discussed by Georganaki et al.. Furthermore, Amin and Hammers reviewed the clinical data of combining various AAT drugs with IT in advanced renal cell cancer patients, where AAT has shown particular benefits owing to high intrinsic VEGF-VEGFR signaling.

Alternative strategies could be employed to enhance T cell infiltration. For example, by performing gene expression analysis to look for correlations to immune profiles, Roszik et al. identified STAT3 as a promising target in cervical cancer. High STAT3 expression was inversely correlated with CD8+ T cell density, implying STAT3 as a promising target to enhance anti-tumor immunity (Roszik et al.). In fact, several clinical trials are investigating STAT3 inhibition. For example, one phase II trial specifically is testing the potential synergy of STAT3 inhibition with anti-PD1 (programmed cell death 1) in colorectal cancer patients (NCT03647839). Another promising approach is specific tumor cell-targeting by utilizing heat-shock-proteins (HSPs), which are overexpressed in various cancers and associated with aggressive phenotypes and poor prognosis (23–26). Circulating levels of HSP70 could serve as prognostic markers (27). HSP70 for instance has been shown to successfully predict response after RT in advanced NSCLC (non-small cell lung cancer) and might serve as a therapeutic target to stimulate anti-tumor natural killer (NK) cell responses (28–30). Indeed, Shevtsov et al. observed a robust increase in infiltrating CD8+ T cells following adoptive transfer of *ex vivo* HSP70-activated NK cells in lung and glioma mouse models. Interestingly, survival benefits were further enhanced by the addition of anti-PD1 therapy (Shevtsov et al.). The exact underlying mechanisms for the described phenotype remain to be determined. However, NK cells can trigger cell death by both apoptosis and necrosis (31), which can lead to activation of the cGAS-STING (cyclic GMP-AMP synthase-stimulator of interferon genes) pathway. The subsequent production of type I IFN has been shown to drive infiltration of CD4+ and CD8+ T cells into tumors (32–34). Although highlighted in the context of RT, Goedegebuure et al. provide a schematic overview of the immune impact of cGAS-STING activation (Goedegebuure et al.: Figure 1). Paradoxically, RT-induced STING activation could also increase MDSCs via CCL2 production, thereby dampening CD8+ T cell activity (Darragh et al.: Figure 2). There are multiple ongoing clinical trials looking at RT and ICB therapies in NSCLC and head and neck squamous cell carcinoma patients, as reviewed by Nardone et al., which will provide important information on how to optimally design the treatment modalities with RT and IT. Interestingly, two recent phase II studies in NSCLC patients looking at adjuvant anti-PD1 therapy after RT, with or without other prior local ablative therapies, reported a promising although non-significant doubling in overall response rates (35) and an impressive increase in progression-free survival (36), thereby highlighting the potential of combining RT with IT.

## Strategy 4: Alleviate Immunosuppression

Tumors are able to employ various resistance mechanisms to evade immune surveillance. The abnormal vasculature is one of the contributors of an immunosuppressive TME (37). The lack of perivascular coverage in tumor blood vessels and high interstitial fluid pressure in tumor tissues often result in malfunctioning or collapsed blood vessels. This results in tumor tissues experiencing high levels of hypoxia, which is one of the main drivers of immunosuppression (38, 39). While tumor cells can readily adapt to the low levels of oxygen, hypoxia also affects the phenotype of stromal cells and immune cells. For example, Tregs and MDSCs have been shown to gain further immunosuppressive capacity (40, 41), and macrophages polarize toward a tumor-promoting phenotype (TAMs) (42) under hypoxic conditions (Figure 6: Darragh et al.). By normalizing the vasculature using AAT, hypoxia can be reduced, and can thereby alleviate immunosuppression (43, 44). The increase in tissue perfusion and oxygenation will also increase the potential impact of RT by optimizing the generation of ROS. As reviewed by Goedegebuure et al., there is a reciprocal relationship where RT, in turn, can have a positive or negative impact on blood vessels and perfusion, depending on the dose and scheduling.

Although vessel normalization by AAT can indirectly improve the immunosuppressive TME, there are ways to directly target and reprogram the immune cells. Focusing on Tregs, Nagai et al. identified PRMT5 (protein arginine methyltransferase) as an interaction partner of FoxP3, a transcription factor important for Treg function. Pharmacological inhibition of PRMT5 led to reduced immunosuppressive activity in Tregs and inhibition of tumor growth (Nagai et al.). Another strategy to reprogram the immune compartment to an anti-tumor phenotype could be to provide IL-2, which would enhance cells such as CD8+ T cells and NK cells (45, 46). However, IL-2 therapy also stimulates Tregs (47), and has been limited by systemic toxicity (48). Mortara et al. reviewed the current efforts of using antibody-cytokine fusion proteins with IL-2 (so-called immunocytokines), designed to be tumor-targeting to overcome these previous limitations and hinder tumor progression by stimulating anti-tumor immunity. In addition to targeting the components of the adaptive immunity, several ongoing trials are investigating the therapeutic benefits of targeting cells of the innate immunity. Specifically, myeloid cells are strong contributors to immunosuppression, especially in glioma, which is the topic covered by Ding et al.. More generally, Dar et al. provided an overview of strategies to target the innate immunity to overcome resistance to RT, with a focus on the interplay between innate and adaptive immunity (see schematic summary in Dar et al.: Figure 1). Furthermore, Menon et al. focused on the stromal contributions to immune evasion and the immunomodulatory properties of RT as an important part of combinatorial treatment modalities. One debated potential effect of RT is the so-called radiation-induced “memory effect” by which prior RT is reported to enhance subsequent anti-tumor immune responses during, for example, ICB therapy. Retrospective analysis by Chen et al. in NSCLC patients suggested that previous RT improved the response to IL-2 infusion, which they attributed to a radiation-induced “memory effect”.

## Strategy 5: Overcome Resistance

As with most therapeutic interventions, intrinsic or acquired resistance is the major obstacle for the success of RT, AAT, and IT. Knowledge of specific tumor-employed resistance mechanisms can offer a strong rational for combinatorial approaches. Darragh et al. discuss several TME-related resistance mechanisms upon RT. For example, RT-induced cell death leads to the release of ATP (adenosine triphosphate), which stimulates DC recruitment and activation (49). However, ATP is catabolized to adenosine by CD39/CD73, which is frequently upregulated on tumor cells and in the TME (50). In contrast to ATP, adenosine is immunosuppressive by limiting DCs and CD8+ T cells, and by simultaneously promoting Tregs and TAMs (Darragh et al.: Figure 4). The review by de Leve et al. highlights the therapeutic potential of targeting CD73/adenosine in cancer to improve RT responses.

To optimally target tumor cells, it has become clear that stem-like (so-called tumor-initiating) cells need to be specifically targeted as they represent a highly resistant population of cells (51, 52). Expression of SDF1 (CXCL12) and its receptor CXCR4 has been linked to stem cell niches where its signaling likely contributes to a stem-like phenotype (53). Hence, RT could greatly benefit from combination strategies with CXCR4-targeting approaches to eliminate resistant clones. The therapeutic potential of such combinations is reviewed by Eckert et al.. Another factor involved in stem cell renewal is TGF-β (transforming growth factor β), which also plays an important role in promoting immunosuppression and fibrosis. Blocking TGF-β by therapeutic antibodies has been shown to slow tumor progression, increase infiltration of T cells and synergize with ICB therapy (54, 55). Rossowska et al. took a different approach in which they modified MC38 tumor cells to secrete exosomes deprived of TGF-β1 (by expressing shRNA) and subsequently using those exosomes as treatment of wildtype MC38 tumors. In doing so, the authors observed a reduction in tumor progression, which was accompanied by increased anti-tumor immunity, thereby highlighting the therapeutic value of targeting TGF-β (Rossowska et al.).

As a concluding remark, antibodies targeting PD1/PD-L1 (programmed cell death ligand 1), with FDA approval in multiple indications have so far shown the most promise in patients. However, resistance is a major hurdle and we are only just beginning to understand the underlying mechanisms. Yao et al. report how anti-PD1 therapy can promote tumor cell proliferation if the tumor cells show intrinsic PD1 expression. In light of such findings, we need to carefully evaluate how to assess PD1/PD-L1 expression before stratifying patients for treatment. Ongoing clinical efforts are indicating that simultaneous targeting of several immune checkpoints, such as CTLA-4 (cytotoxic T-lymphocyte-associated antigen-4), Lag-3 and Tim-3, could offer significant advantages over single ICB therapies. Khair et al. provide an exhaustive overview on this topic.

## Summary

One major concern when treating patients with ICBs, such as anti-PD1 and anti-CTLA-4 antibodies, is the high frequency of immune-related adverse events. This, along with lacking a reliable biomarker for patient stratification, underscores the need for multimodal therapy allowing for the use of lower doses and implementation of standard operating procedures to manage these side-effects without compromising efficacy. However, as is evident throughout the contributions in this article collection, several important outstanding questions remain to be fully addressed including optimal dosage, timing, and scheduling for these combinatorial approaches.

## Author Contributions

Both authors have actively participated in shaping the idea for the article collection, recruiting authors, and acting as editors for several of the contributions. PA and CO wrote the editorial together and made final edits.

### Conflict of Interest Statement

The authors declare that the research was conducted in the absence of any commercial or financial relationships that could be construed as a potential conflict of interest.
